# Effect of co-morbidities on disease course in human immunodeficiency virus-infected illicit drug users in the era of highly active antiretroviral therapy

**DOI:** 10.4178/epih/e2015008

**Published:** 2015-02-18

**Authors:** Venkataramana Kandi

**Affiliations:** Department of Microbiology, Prathima Institute of Medical Sciences, Karimanagr, India

Human immunodeficiency virus (HIV), the causative agent of acquired immunodeficiency syndrome (AIDS), has been responsible for severe morbidity and mortality worldwide. Since its discovery in 1980, HIV infection has assumed prominent significance among other prevalent infectious diseases worldwide. It has been very difficult for physicians and health care workers to promote public understanding of HIV infection because of social proscriptions of some behaviors associated with HIV; however, there have been significant developments in the scientific understanding of the causative virus, modes of transmission, laboratory diagnosis and treatment of HIV infection in the last decade. HIV infection alone has been noted to influence haematological [1] and biochemical parameters [2], leading to anaemia, lymphocytopaenia [3], and liver and cardiovascular abnormalities [4,5]. The initiation of highly active antiretroviral therapy (HAART) in individuals with these symptoms may contribute to further worsening of the disease and result in further morbidity and mortality [6,7]. The disease course of HIV is influenced by the presence of co-morbidities that include infectious diseases like tuberculosis (TB), hepatitis B virus (HBV), and hepatitis C virus (HCV) as well as malignancies. Other factors that influence disease management and antiretroviral therapy in the HIV-infected population are illicit drug use and malnourishment. Therefore, identification of various co-morbidities prior to the initiation of HAART is necessary to minimise related additional complications and resultant morbidity and mortality.

Illicit drug users are among the individuals most predisposed to contract HIV and other blood-borne infectious agents. There is not much data available globally on the influence of co-morbidities and treatment challenges faced when treating HIV-positive individuals with comorbid substance use disorders.

Addiction is defined as a psychological condition in which an individual has a craving for a substance and uses the substance compulsively and repeatedly in spite of knowing its harmful effects [8]. Alcohol and nicotine are among the most commonly abused substances worldwide due to their availability and affordability. Other substances of abuse are cocaine, heroin, opium, ketamine, methamphetamine and many others. The HIV-positive population has been noted to have a higher chance of developing drug abuse and infectious diseases than HIV-negative individuals, which could be attributed to behavioural (sharing injecting equipment), sociocultural (marginalisation and stigmatisation of HIV-positive people), environmental (reduced accessibility to healthy living conditions, limited access to sterile syringes), and infrastructural factors (minimal access to addiction treatment programmes) that increase the chance of disease transmission [9]. Moreover, illicit drug users with comorbid HIV infection have a greater chance of developing complications from antiretroviral therapy than drug users without HIV infection. HIV-positive illicit drug users, compared with HIV-positive individuals, have significantly higher chances of developing other infections like tuberculosis [10], HBV and HCV [11,12], herpes simplex virus and many others including sexually transmitted diseases [13] like syphilis [14] and gonorrhoea.

Recent studies have reported that increased disease burden [15], mental illness [16], cause-specific disability-adjusted life years and years lost because of disability are significantly related to illicit drug use [17]. Studies have also observed that substances of abuse may enhance viral replication and reduce TCD4+ cell count and thereby hasten the progression of HIV-related disease [18]. Illicit drug use has been noted to predispose individuals to infection with HIV (9 to 12%), HCV (50%) and hepatitis A virus (2%) in the US [19]. Comorbidities in HIV-infected illicit drug users may vary in different geographical regions, as evidenced by an Indian study that noted a greater prevalence of oral candidiasis (43.2%), TB (33.9%) and anaemia (22%) among this population [20]. Considering that the majority of patients diagnosed as HIV-seropositive are in their most productive years of life, a better knowledge of the prevalence of co-morbidities and substance abuse in such individuals assumes significance for better patient care [21,22].

Therefore, identification of patients with HIV infection and associated co-morbidities, including substance abuse, becomes very important for improved clinical management. Strengthening of primary care for the HIV-infected population should be considered a first step towards better HIV disease management. The voluntary counselling and testing program which is available in most parts of the world where HIV is prevalent for those using illicit drugs should be revitalised to promote early diagnosis and better understanding of substance abuse disorders. Identification of the factors contributing to substance abuse in HIV-positive patients and planning strategies for treatment of addiction, as well as motivating patients to undertake antiretroviral therapy with regular follow-up would contribute to improved quality of life in such individuals.

Public health strategies such as vaccination, pre-vaccination testing programmes, outreach initiatives to engage high risk populations, regular monitoring for treatment adherence, and peer counselling about the adverse effects of substance abuse on the HIV disease course and antiretroviral therapy would also contribute to improved management of HIV seropositive patients. Finally, education about the importance of safe sex practices and sterile injection practices, facilitating self-assessment of risk for contracting infectious diseases, and promoting participation in treatment programmes for substance abuse will synergistically be instrumental in the management of HIV-seropositive patients using illicit drugs and in reducing morbidity and mortality and increasing quality of life in the era of HAART.

In conclusion, there is a pressing need for epidemiologial studies on the prevalence of co-morbidities among HIV-infected illicit drug users and the formulation of strategies to provide effective health care in these patients.
